# Bioluminescent Imaging of Genetically Selected Induced Pluripotent Stem Cell-Derived Cardiomyocytes after Transplantation into Infarcted Heart of Syngeneic Recipients

**DOI:** 10.1371/journal.pone.0107363

**Published:** 2014-09-16

**Authors:** Vera Lepperhof, Olga Polchynski, Klaus Kruttwig, Chantal Brüggemann, Klaus Neef, Florian Drey, Yunjie Zheng, Justus P. Ackermann, Yeong-Hoon Choi, Thomas F. Wunderlich, Mathias Hoehn, Jürgen Hescheler, Tomo Šarić

**Affiliations:** 1 Institute for Neurophysiology, Medical Faculty, University of Cologne, Cologne, Germany; 2 In-vivo-NMR Laboratory, Max Planck Institute for Neurological Research, Cologne, Germany; 3 Department of Cardiothoracic Surgery, Heart Center of the University of Cologne, Cologne, Germany; 4 Center for Molecular Medicine Cologne, University of Cologne, Cologne, Germany; 5 Max Planck Institute for Metabolism Research and Institute for Genetics, Cologne, Germany; 6 Cologne Cluster of Excellence in Cellular Stress Responses in Aging-Associated Diseases (CECAD), Cologne, Germany; University of Minnesota Medical School, United States of America

## Abstract

Cell loss after transplantation is a major limitation for cell replacement approaches in regenerative medicine. To assess the survival kinetics of induced pluripotent stem cell (iPSC)-derived cardiomyocytes (CM) we generated transgenic murine iPSC lines which, in addition to CM-specific expression of puromycin *N*-acetyl-transferase and enhanced green fluorescent protein (EGFP), also constitutively express firefly luciferase (FLuc) for bioluminescence (BL) *in vivo* imaging. While undifferentiated iPSC lines generated by random integration of the transgene into the genome retained stable FLuc activity over many passages, the BL signal intensity was strongly decreased in purified iPS-CM compared to undifferentiated iPSC. Targeted integration of FLuc-expression cassette into the ROSA26 genomic locus using zinc finger nuclease (ZFN) technology strongly reduced transgene silencing in iPS-CM, leading to a several-fold higher BL compared to iPS-CM expressing FLuc from random genomic loci. To investigate the survival kinetics of iPS-CM *in vivo*, purified CM obtained from iPSC lines expressing FLuc from a random or the ROSA26 locus were transplanted into cryoinfarcted hearts of syngeneic mice. Engraftment of viable cells was monitored by BL imaging over 4 weeks. Transplanted iPS-CM were poorly retained in the myocardium independently of the cell line used. However, up to 8% of cells survived for 28 days at the site of injection, which was confirmed by immunohistological detection of EGFP-positive iPS-CM in the host tissue. Transplantation of iPS-CM did not affect the scar formation or capillary density in the periinfarct region of host myocardium. This report is the first to determine the survival kinetics of drug-selected iPS-CM in the infarcted heart using BL imaging and demonstrates that transgene silencing in the course of iPSC differentiation can be greatly reduced by employing genome editing technology. FLuc-expressing iPS-CM generated in this study will enable further studies to reduce their loss, increase long-term survival and functional integration upon transplantation.

## Introduction

Heart failure is the most frequent cause of death in adults in developed countries [1]. Cellular replacement approaches hold great promise for the treatment of this disease. Embryonic stem cells (ESC) [2], [3], induced pluripotent stem cells (iPSC) [4], [5], parthenogenetic stem cells [6] and their cardiomyocyte (CM) derivatives [7]–[12], as well as cardiac precursor cells [13], [14] and autologous adult stem cells [15], [16], have been shown to exert beneficial effects on the function of the injured heart after transplantation. However, only contractile CM are capable of electromechanical coupling with the host heart tissue, thereby contributing to its pump function [9], [12], [17]. Among different types of stem cells, iPSC represent an attractive source of contractile CM for cardiac repair. These cells can be obtained from autologous sources, are ethically unobjectionable and similar to ESC-derived CM (ES-CM) in regard to their molecular [18], structural [19] and functional [20], [21] properties. Furthermore, like ES-CM, transplanted purified murine iPS-CM can also improve the contractile heart function following chronic myocardial infarction in animal models of this disease [11].

Despite great promises of cell-based therapeutic strategies for damaged heart, studies on integration efficiency and therapeutic benefit of cells transplanted into the myocardium have been greatly impeded by the loss of cells after cardiac delivery [22]–[28]. The most important factors responsible for this phenomenon are cell death due to ischemia and inflammation at the site of injection, mechanical loss of cells immediately after delivery and lack of functional coupling of transplanted cells with host CM [27]. In order to determine the number of cells successfully delivered into the site of injury and develop strategies for improving their engraftment and survival, approaches for quantitative *in vivo* monitoring of viable cells after transplantation are required. A number of methods such as quantitative PCR, magnetic resonance imaging (MRI), radionuclide imaging (e.g. positron emission tomography (PET) and single photon emission computed tomography (SPECT) and reporter gene imaging (e.g. bioluminescence (BL) imaging) have been employed for this purpose (reviewed in [29]). BL imaging has been extensively used in preclinical models for longitudinal *in vivo* monitoring of various types of transplanted stem cells [16], [22], [23], [26], [30], [31]. Although this method requires the genetic modification of cells to express one of the luciferase enzymes, has a low spatial resolution and is not translational to humans, it enables affordable, highly sensitive, non-radioactive detection and quantification of viable cells in live small animals. BL imaging was applied by several groups to track the *in vivo* engraftment of ES-CM [22], [23], [31]. However, careful analysis of the survival kinetics of iPS-CM after transplantation into the infarcted heart of syngeneic recipients using BL imaging has not yet been performed.

In the present study, we established a transgenic iPSC line constitutively expressing firefly luciferase (FLuc) as well as the antibiotic resistance gene puromycin N-acetyl-transferase (PAC) and EGFP under control of the cardiac specific alpha-myosin heavy chain (αMHC) gene promoter allowing for isolation, visualization and *in vivo* tracking of purified iPS-CM. The expression of FLuc was driven by various constitutive promoters (Ubiquitin C, CAG) after random genomic integration of the transgene in iPSC. An additional ESC line in which FLuc expression was driven by the phosphoglycerate kinase (PGK) promoter was also generated. All established cell lines exhibited stable FLuc activity in the course of *in vitro* expansion. However, upon initiation of differentiation strong silencing of FLuc expression was encountered in all cell lines independently of the promoter used. The transgene silencing was minimized by insertion of the UbC promoter-driven FLuc cassette into the ROSA26 safe harbor locus using zinc finger nuclease (ZFN)-based genome editing [32]. We selected one iPSC line generated by ZFN approach and one generated by random insertion of the transgene, in which pUbC-driven FLuc activity was high enough in purified CM to ensure their *in vivo* detection after transplantation into the periinfarct region of cryoinjured hearts of syngeneic recipients. Intramyocardially transplanted FLuc-ROSA iPS-CM exhibited four-fold higher BL signal intensity than iPS-CM expressing FLuc from random loci. However, in both iPSC lines the majority of CM were lost within 7 days after injection. Nevertheless, despite the initial cell loss, isolated patches of surviving iPS-CM could still be found in histological sections of the myocardium 28 days post transplantation. These data are in agreement with those reported for ES-CM and other cell types and indicate that despite the long-term survival of a minority of CM, efforts must be undertaken to improve their retention and survival after transplantation in order to maximize their therapeutic efficacy.

## Methods

### Ethics statement

All animal experiments described in this study were approved by the Landesamt für Natur, Umwelt und Verbraucherschutz NRW, 45659 Recklinghausen, Germany (Permit Number: 8.86–50.10.37.09.161) and conformed to the Directive 2010/63/EU of the European Parliament. All efforts were made to minimize suffering of animals.

### Generation of firefly luciferase expression vectors

Firefly luciferase expression vectors used in this study was generated by inserting either the ubiquitin C (UbC), phosphoglycerate kinase (PGK) or chicken β-actin promoter with CMV enhancer (CAG) into the multiple cloning site of the promoterless pGL4.14 [*luc2*/Hygro] plasmid (Promega, Madison, WI). UbC promoter (pUbC) was amplified from plasmid pLenti6/UbC/V5-DEST (Invitrogen, Life Technologies, Carlsbad, CA) with Dream Taq PCR Master Mix (Fermentas, Thermo Scientific, Waltham, MA) using primers flanked by restriction sites for *Xho*I and *Hind*III (Fermentas). pGL4.14 [*luc2*/Hygro] plasmid and the PCR-product were digested with both enzymes, purified with NucleoSpin Gel and PCR Clean-up-Kit (Macherey-Nagel, Düren, Germany) and ligated overnight at 4°C with Rapid DNA Ligation Kit (Fermentas) to yield the pGL4.14-pUbC [*luc2*/Hygro] vector. The PGK promoter was removed from the pPPGKIP vector by digestion with *Sal*I and *Hind*III and subcloned between the XhoI and HindIII cutting sites of the pGL4.14 [*luc2*/Hygro] plasmid to obtain pGL4.14-pPGK [*luc2*/Hygro] vector. In order to generate plasmid in which FLuc expression is controlled by the CAG promoter, the CAG promoter sequence was isolated from plasmid pCAG-DsRed (Addgene plasmid 11151, [33]) with *Sal*I and ligated into the plasmid pCDNA3.1-CMV-CD4-IRES-FLuc from which the CMV-promoter sequence was first removed by digestion with the isoschizomer *Xho*I. The CAG promoter in the resulting plasmid pCDNA3.1-pCAG-CD4-IRES-FLuc drives the expression of truncated human CD4 receptor (lacking its intracellular domain) and IRES-flanked FLuc. The detection of CD4 by flow cytometry on the surface of transgenic cells enabled additional means for monitoring the CAG promoter activity in undifferentiated iPSC and their derivatives at different stages of differentiation. In order to enable antibiotic selection of stably genetically modified cells, the entire CAG-CD4-IRES-FLuc cassette from pCDNA3.1-pCAG-CD4-IRES-FLuc plasmid was excised with *Cla*I and *Spe*I and ligated into *Cla*I and *Spe*I digested pGL4.14 [*luc2*/Hygro] plasmid (which removed the original luc2 sequence) to yield the pGL4.14-pCAG [*CD4-IRES-FLuc*/Hygro] plasmid. All ligation-products were used for transformation of *E. coli* and clones containing the correct insert were identified by colony-PCR. Plasmids were isolated using PureLink HiPure Plasmid Miniprep Kit (Invitrogen), validated by sequencing and used for electroporation of murine iPSC.

### Establishment of stable transgenic firefly luciferase-expressing iPSC lines

In this study, we have used the murine iPSC line TiB7.4 that was generated from tail tip fibroblasts isolated from 129S4/Sv4JaeJ x C57BL/6 mice and was kindly provided by R. Jaenisch and A. Meissner [34]. Cells were first electroporated with a previously described plasmid vector αPIG [10] containing the **P**AC-gene and **I**RES-flanked E**G**FP-gene under the control of the cardiac specific alpha-myosin heavy chain (**α**MHC) gene promoter (GenBank Accession No. U71441) to generate the stable transgenic **αPIG**-iPSC line enabling isolation of pure iPS-CM (Fatima A. et al., *manuscript in preparation*). The FLuc expression plasmid pGL4.14-pUbC [*luc2*/Hygro] was then introduced into the αPIG-iPSC line (clone AT25) by electroporation. Plasmid was linearized with *Sal*I and 5 µg were added to 5×10^6^ cells in 1 ml phosphate buffered saline (PBS). The cells were electroporated using a Gene Pulser (BioRad, Hercules, CA, USA) at 250 V and 500 µFd in a 0.4 cm gap width mammalian cell cuvette (BioRad). The αPIG-iPSC line expressing FLuc and CD4 from CAG promoter was generated by nucleofection of 50,000 cells with 400 ng of *Spe*I-linearized pGL4.14-pCAG [*CD4-IRES-FLuc*/Hygro] plasmid in 20 µl of iPSC-specific nucleofection solution in a 96-well nucleofection cuvette placed in a 4D-Nucleofector (Lonza, Cologne, Germany). In addition to the above described iPSC lines, we have also generated murine ESC lines expressing FLuc under the control of the PGK promoter. For this purpose the αPIG-ESC line that was described previously [10] was electroporated with *Pst*I-linearized pGL4.14-pPGK [*luc2*/Hygro] vector following the above described procedure. Immediately after pulsing or nucleofection, transfected cells were transferred at different densities (0.5×10^6^ or 4.5×10^6^ cells per plate for electroporated cells and 5×10^4^ cells per plate for nucleofected cells) onto 10 cm dishes previously seeded with 1×10^6^ hygromycin-resistant murine embryonic fibroblasts (MEF). Successfully transfected cells were selected with 50 µg/ml hygromycin (InvivoGen, San Diego, CA, USA) with medium change every 48 hours. After 10 days, hygromycin resistant pUbC-FLuc and pCAG-CD4/FLuc αPIG-iPSC-colonies and pPGK-FLuc αPIG-ESC-colonies were picked into a 96-well format seeded with hygromycin-resistant MEF (1.5×10^4^ cells per well), expanded, tested for BL intensity and either cryopreserved or used for further experiments.

### Construction of a donor plasmid for transgene insertion into the ROSA26 locus

The DNA fragment containing UbC promoter driving constitutive expression of FLuc gene and SV40-promoter driving expression of a selectable marker for hygromycin resistance was isolated from pGL4.14-pUbC [*luc2*/Hygro] vector by digestion with restriction enzymes *Xho*I and *Sal*I (Fermentas). The fragment was then ligated into the *Xho*I and *Sal*I double digested pDonor-MCS-ROSA26 plasmid (Addgene Plasmid 37200, [35]), resulting in the plasmid pDonor-pUbC [*luc2*/Hygro]-ROSA26 (referred to hereafter as pUbC-FLuc-ROSA) (**Figure S1** in File S1). Plasmid isolation and validation were performed as described above.

### Targeted insertion of FLuc into ROSA26 locus of murine iPSC

In brief, 2×10^5^ αPIG-iPSC were washed in PBS, centrifuged and resuspended in 10 µl R buffer (Neon Transfection System, Invitrogen) with a total of 4 µg of pUbC-FLuc-ROSA donor plasmid and 500 ng of each of the ZFN-encoding plasmid pCMV-RosaL6 and pCMV-RosaR4 that target the first intron of the ROSA26 locus [35] (**Figure S1** in File S1). The cells were transfected with the Neon transfection system at 1400 V, 10 ms and 3 pulses. Cells were then immediately plated on 6 cm plates containing 0.8×10^6^ hygromycin resistant MEF. Hygromycin selection (50 µg/ml) was started at day 5 after transfection. After a week of selection, individual clones were picked, expanded and characterized as described above.

### Validation of ZFN-edited iPSC by Southern blot and PCR

In order to verify the correct insertion of a transgene into the ROSA26 locus, 20 µg of genomic DNA isolated from pUbC-FLuc-ROSA iPSC clones using the DNeasy Blood & Tissue kit (Qiagen) was digested with *Eco*RI, separated on a 0.8% agarose gel and transferred to a nylon membrane (Amersham Hybond XL, GE Healthcare Buckinghamshire, GB). Southern blot analysis was performed by using an external 1 kb probe located in the ROSA26 locus upstream of the transgene integration site that was isolated by BamHI-EcoRI digestion from a plasmid (A03) provided by Mao et al. [33] and labeled using α-^32^P-dCTP and the Ladderman Labeling Kit (Takara, Otsu, Japan). This probe detects the wild-type and targeted ROSA26 allele with sizes of 15630 and 4066 nt, respectively (**Figure S1** in File S1). The integration of targeting construct into the ROSA26 locus was also verified by PCR using the forward primer binding to donor vector and reverse primer binding to ROSA26 locus downstream of the integration site (**Figure S1** and **Table S1** in File S1). The cycling program was: 94°C for 2 min followed by 40 cycles of 94°C for 35 s, 60°C for 45 s, and 72°C for 65 s. PCR was performed with DreamTaq PCR Mastermix (Fermentas) using 200 ng of genomic DNA as a template. The presence of PCR product with the expected size of 950 bp was analyzed by agarose gel electrophoresis and visualized by staining with ethidium bromide.

### Cell culture and cardiac differentiation

All iPSC and ESC lines were cultured in DMEM supplemented with 15% fetal bovine serum (FBS), 1× non-essential amino acids (NEAA), 100 µM β-mercaptoethanol (β-ME) and 1000 U/ml leukemia inhibitory factor (LIF) (Merck Millipore, Darmstadt, Germany) on murine MEF and passaged every 2–3 days with a seeding density of 0.4×10^6^ cells per 6 cm-dish containing 0.8×10^6^ MEF. For cardiac differentiation, 2×10^6^ cells were transferred into bacterial dishes containing differentiation medium composed of IMDM supplemented with 20% FBS, 1x NEAA and 100 µm β-ME to induce embryoid body (EB) formation in a dynamic suspension culture on a horizontal shaker. After two days, 3×10^4^ EB were transferred into a 250 ml spinner flask (CELLspin 250, IBS Integra Biosciences, Fernwald, Germany) containing 200 ml of differentiation medium. At day 9 of differentiation, 50 ml of medium was replaced by fresh medium, and 8 µg/ml of puromycin (InvivoGen) was added to allow for selection of CM. On day 12 of differentiation, EB were transferred to 10 cm bacterial plates and further selected with puromycin for additional 3–4 days. For transplantation and functional analyses, the purified cardiac clusters were dissociated into single CM with 0.25% trypsin-EDTA supplemented with 5 U/ml of DNAse I (Sigma-Aldrich, St. Louis, MO, USA). If not indicated otherwise, all cell culture media components were purchased from Invitrogen, Life Technologies.

### Flow cytometry

Dissociated GFP-expressing CM were suspended in PBS without calcium and magnesium, supplemented with 0.2% FBS and analyzed with a BD FACScan (BD Pharmingen, Franklin Lakes, NJ, USA). 2×10^4^ events were acquired. Dead cells were identified after propidium iodide (PI) addition and excluded by proper gating. Viable cells were then quantified for expression of EGFP by quadrant statistical analysis using Cyflogic freeware (http://www.cyflogic.com/). The expression of CD4 on the surface of CAG-CD4/FLuc αPIG-iPSC was determined by staining with a PE-conjugated antibody (cat. no. 555347, BD Pharmingen, Heidelberg, Germany) according to the manufacturer's recommendations. Control cells were stained with an isotype control antibody (Santa Cruz Biotechnology, Santa Cruz, CA). Quantitative analysis of the data was performed with the Cyflogic freeware.

### Measurement of bioluminescence in the microplate reader

For luminescence measurements, cells were centrifuged and lysed by resuspending in 50 µl BrightGlo-Buffer (Promega, Madison, WI, USA). Protein concentration was determined by Bradford assay (Roth, Karlsruhe, Germany). For BL intensity-measurement, 5 µg of protein lysate were measured in triplicates with the BrightGlo Kit from Promega in a GeniosPro microplate reader (Tecan, Crailsheim, Germany) in white flat-bottom 96-well plates (Corning Life Sciences, Tewksbury, MA, USA) according to the manufacturer's recommendations.

### Promoter activity assay

The activity of the UbC promoter in pGL4.14 [*luc2/Hygro*] plasmid was determined in undifferentiated iPSC and purified iPS-CM. CM were differentiated from αPIG-iPSC and purified by puromycin selection. At day 16 of differentiation, the purified αPIG-iPSC-derived cardiac clusters were dissociated by 0.25% trypsin-EDTA and single CM seeded on plates coated with 2.5 µg/ml fibronectin (Sigma-Aldrich). 1×10^5^ plated iPSC or CM were co-transfected with 1 µg of pGL4.14-pUbC [*luc2*/Hygro] expression plasmid and 1 µg of the renilla luciferase-encoding plasmid pRL-SV40P (Addgene plasmid 27163, [36]) as an internal control for transfection efficiency using Lipofectamine 2000 in OptiMEM (Invitrogen) according to the manufacturer's protocol. After 24 hours, cells were lysed with BrightGlo-Buffer as described above and luminescence of FLuc and renilla luciferase measured in the plate reader using the DualGlo-Kit (Promega).

### Cell transplantation

For transplantation, defined numbers of FLuc-expressing iPS-CM were suspended in PBS containing calcium chloride and magnesium chloride. Male C57/BL6 mice were anesthetized with 3% isoflurane (Baxter, Unterschleißheim, Germany) in the absence of muscle relaxants, placed onto a heating plate warmed to 38°C, intubated and ventilated with a mixture of nitrous oxide and oxygen (1∶1) and 1.25% isoflurane at a rate of 130 heaves per minute with a volume of 0.5 to 0.8 ml, depending on the size of the animal. The mice were shaved at the injection site, and after performing a skin incision, the muscles were loosened and the thoracic cavity was opened by inserting a retractor into the intercostal space between the 3^rd^ and 4^th^ rib. The heart was exposed, the left ventricular myocardium was cryoinjured and 5 µl of cell suspension injected into the single peri-infarct region using a 25 µl Hamilton syringe (Model 702 RN SYR, Hamilton, Bonaduz, CH) and a 27G needle. Postoperative analgesia was provided by s.c. administration of Tramadol at 15 mg/kg after the extubation and at 1 mg/ml in the drinking water for 4 additional days (Gruenenthal, Aachen, Germany). For transplantations into the hind leg skeletal muscle, the dissociated UbC-FLuc or UbC-FLuc-ROSA iPS-CM were suspended in PBS and injected as described above.

### Whole body and whole cell bioluminescence imaging

Prior to imaging, mice were injected intraperitoneally with 300 mg D-luciferin (Caliper Life Sciences, Hopkinton, MA, USA) per kg body weight in PBS. Ten minutes after substrate injection, the mice were anesthetized with 2.5% isoflurane and imaging was performed in an IVIS 200 system (Xenogen, Caliper Life Sciences) for 60 seconds at high binning. BL signal was quantified in the region of interest (ROI) with the software “Living Image 3D”, version 2.5.1. For experiments in which the BL signal intensity of undifferentiated FLuc-iPSC and purified FLuc-iPS-CM was determined *in vitro* prior to transplantation, different numbers of serially diluted cells were resuspended in PBS containing 15 mg/ml D-luciferin, 5 µl were transferred into black round-bottom 96-well plates and the BL intensity was measured in the IVIS after 10 min incubation with the same settings as described above.

### Immunohistochemistry

After final BL measurements, mice were euthanized, hearts excised, flushed with PBS, embedded in Tissue-Tek O.C.T. (Sakura Finetek, Staufen, Germany) and snap-frozen in isobutane (2-methylpropane, Roth) cooled with dry ice and stored at −80°C. Hearts were cryo-sectioned (10 µm) along the short axis (transversal) using a cryotome CM-1950 (Leica, Wetzlar, Germany). Sections were postfixed for 5 minutes in 4% paraformaldehyde and stained with primary antibodies for cardiac α-actinin (Cat. No. A7811, Sigma-Aldrich) diluted 1∶800 and EGFP (Cat. No. A11122, Molecular Probes, Eugene, USA) diluted 1∶200. Fluorescent secondary antibodies conjugated to Alexa Fluor 594 (Molecular Probes) were used to detect primary antibodies at the dilution of 1∶1000. Cell nuclei were visualized with Hoechst 33342 (Life Technologies) and cover slips were embedded using 10 µl mounting medium Prolong Gold (Life Technologies). Microphotographs were taken using a confocal laser scanning microscope (Olympus, FluoView FV1000, Tokyo, Japan).

## Results

### BL intensity of pUbC-FLuc αPIG-iPSC decreases during spontaneous differentiation

**Figure 1:**
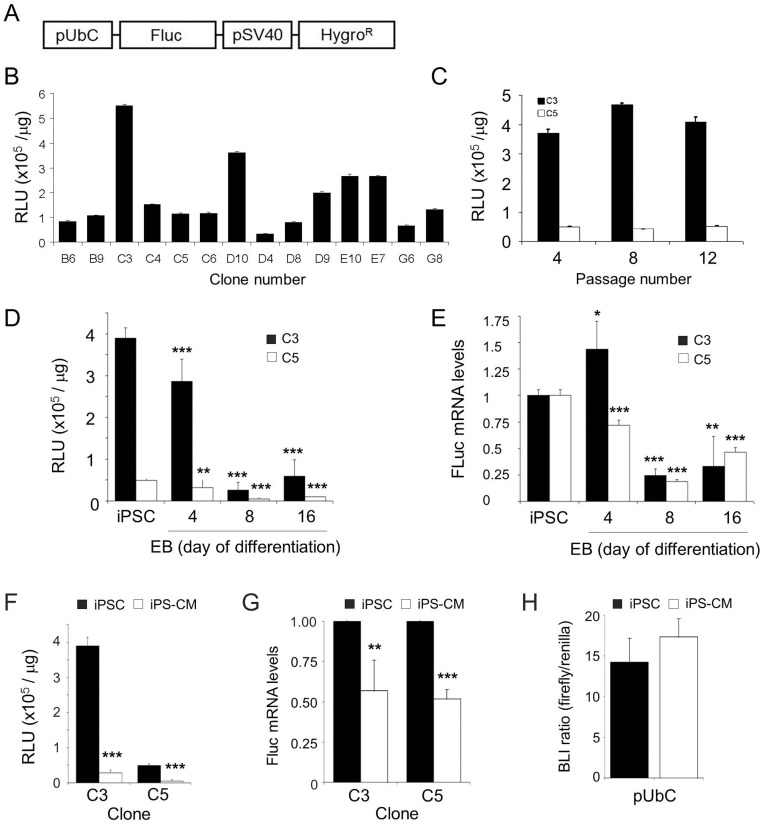
Luciferase activity in stable FLuc-αPIG-iPS cells is clone dependent and decreases after spontaneous differentiation. **A.** Scheme of the pGL4.14-pUbC [*luc2/Hygro*] plasmid used to generate stable iPSC lines constitutively expressing FLuc. **B.** BL signal intensity in protein lysates (5 µg/well) of 14 hygromycin-resistant pUbC-FLuc iPSC clones measured in triplicates using the BrightGlo Kit in a microplate reader. Data are shown as relative luminescence units (RLU) per µg of protein (n = 3). **C.** The stability of FLuc activity in transgenic pUbC-FLuc iPSC clones C3 and C5 over prolonged *in vitro* expansion (n = 3). **D.** BL signal intensity in protein lysates of pUbC-FLuc EB collected at different days during spontaneous differentiation as determined in microplate reader (n = 6 for C3, n = 9 for C5). **E.** Relative levels of FLuc transcript expression during spontaneous differentiation of pUbC-FLuc iPSC as determined by RT-qPCR (n = 6 from two independent differentiations). **F.** BL signal intensity in two clones (C3 and C5) of undifferentiated pUbC-FLuc iPSC and puromycin purified pUbC-FLuc iPS-CM on day 16 of differentiation. Data are given as mean ± SD of 2 independent experiments for clone C3 and 3 for clone C5, each measured in technical triplicates. **G.** Relative FLuc-transcript expression in two clones of purified pUbC-FLuc iPS-CM relative to undifferentiated pUbC-FLuc iPSC as determined by RT-qPCR. Data are shown as mean ± SD of 2 independent experiments measured in technical triplicates. **H.** UbC promoter activity in native iPSC and purified native iPS-CM measured 24 hours after transient transfection with pGL4.14-pUbC [*luc2/Hygro*] plasmid and renilla luciferase-encoding control plasmid. Values shown are ratios of FLuc activity relative to co-transfected renilla lucifderase activity given as mean ± SD (n = 3), p>0.05. Statistical analyses were performed by two-tailed paired Student's t-test: *p<0.05; **p<0.01; ***p<0.001.

To enable *in vivo* BL tracking of transplanted iPS-CM, a genetically modified iPSC line expressing EGFP and puromycin resistance under the cardiac specific αMHC promoter (Fatima A. et al., *manuscript in preparation*) was transfected with the expression plasmid containing firefly luciferase gene under the control of the constitutive UbC promoter and the antibiotic resistance gene hygromycin B phosphotransferase regulated by the SV40 promoter (**Figure 1A**). Ninety-six pUbC-FLuc αPIG-iPSC-clones were selected ten days after initiation of the hygromycin selection and tested *in vitro* for FLuc activity. Fourteen clones that showed various levels of BL intensity (**Figure 1B**) were expanded for further testing. Of those, the iPSC clones C3 and C5 retained stable FLuc activity over 12 passages (**Figure 1C**) and expressed pluripotency markers alkaline phosphatase, Oct4 and SSEA1 at levels similar to the parental iPSC line (data not shown). When these clones were subjected to spontaneous differentiation over 16 days, the FLuc activity strongly decreased in EB of both analyzed clones with similar kinetics (**Figure 1D**). The slight loss of FLuc activity in EB cells on day 4 of differentiation was followed by massive reduction of FLuc intensity on day 8. By termination of the experiment on day 16 of differentiation 15.2±10.1% (clone C3) and 19.9±3.0% (clone C5) of the original FLuc activity in the pluripotent state of each respective clone was measured (**Figure 1D**). The decrease of FLuc mRNA expression levels in EB of these two clones was less pronounced, but followed a pattern similar to that observed for the BL activity. At day 16 of differentiation, the FLuc transcript levels in EB cells of clone C3 and C5 decreased, respectively, to 33.1±28.2% and 46.5±4.5% of the original FLuc mRNA levels in iPSC (**Figure 1E**).

### FLuc activity in purified UbC-FLuc iPS-CM *in vitro*

Our functional analyses revealed that genetic modification of iPSC did not alter their cardiac differentiation capacity and that drug selected pUbC-FLuc αPIG-iPS-CM displayed normal structural and functional properties (**Supplemental data** and **Figure S2** in File S1). We next determined the BL activity in purified CM derived from clones C3 and C5. The BL intensity in CM lysates decreased in two independent differentiation experiments to 7.4±1.7% and 10.8±7.5% of the initial BL activity in FLuc-αPIG-iPSC, respectively (**Figure 1F**). In order to determine the FLuc mRNA levels qPCR was performed. Interestingly, the decrease at the FLuc transcript level was less pronounced compared to the decrease in BL activity. Relative to the FLuc mRNA levels in undifferentiated iPSC, the expression of FLuc mRNA in purified CM derived from iPSC clones C3 and C5 was diminished to 57.0±18.8% and 51.8±5.7% of the levels in iPSC, respectively (**Figure 1G**). The observed reduction of BL activity and FLuc transcript levels during differentiation could be due to differences in the ability of the transcriptional machinery of undifferentiated iPSC and purified CM to activate the UbC promoter driving the expression of FLuc. To address this question, we assessed the pUbC activity in parental non-luminescent αPIG-iPSC and purified CM derived from them. Cells were co-transfected with the pUbC-pGL4.14 [*luc2/Hygro*] plasmid and a renilla luciferase expression plasmid pRL-SV40P that served as a transfection control. No differences in UbC promoter activity between undifferentiated iPSC and purified CM were detected 24 hours after transfection (**Figure 1H**), suggesting that the reduction of Fluc activity in CM resulted from epigenetic silencing of the stably integrated vector during differentiation of iPSC.

### FLuc silencing occurs during spontaneous differentiation independently of the promoter used

**Figure 2:**
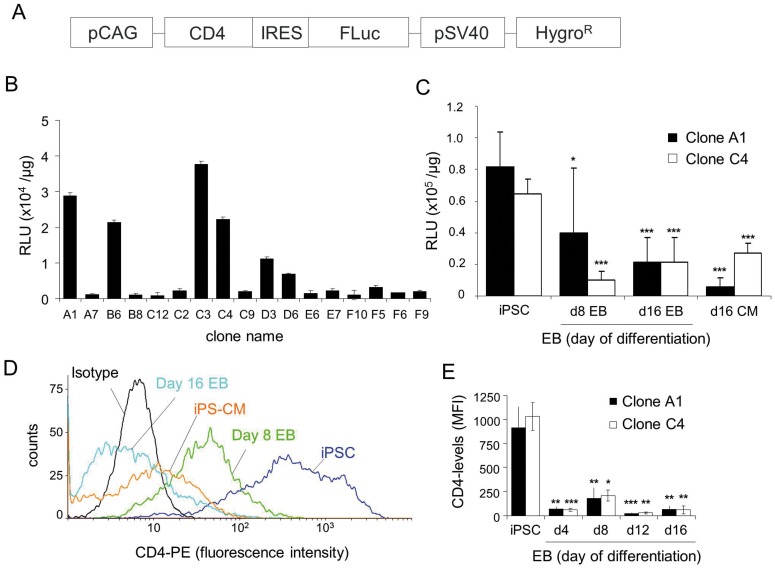
Luciferase activity and CD4 coreceptor expression in stable pCAG-CD4/FLuc iPSC clones decrease during differentiation *in vitro*. **A.** Schematic representation of the plasmid used to generate transgenic cell line expressing FLuc and extracellular domain of CD4 receptor under the control of CAG-promoter. **B.** FLuc-activity in 17 pCAG-CD4/FLuc αPIG-iPSC clones after hygromycin selection. BL was measured in iPSC lysates in a luminescence plate reader (n = 3). Values are given as mean relative luminescent units (RLU) ± SD per µg protein. **C.** FLuc-activity of the two pCAG-CD4/FLuc αPIG-iPSC clones was measured in cell lysates before and after spontaneous *in vitro* differentiation and selection of cardiomyocytes (d16 CM). Values are given as mean RLU ± SD per µg protein (n = 3). *p<0.05; ***p<0.001. **D.** Flow cytometric analysis of CD4 expression on the surface of pCAG-CD4/FLuc iPSC clone C4 in pluripotent state, day 8 EB, day 16 EB and puromycin selected CM at day 16 of differentiation. **E.** Quantification of CD4 expression levels in pCAG-CD4/FLuc iPSC clones A1 and C4 at different stages of differentiation. Values are given as mean fluorescent intensity (MFI) ± SD of triplicate measurements. *p<0.05; **p<0.01, ***p<0.001, compared to MFI of CD4 expression in iPSC.

To determine whether the decline of Fluc activity in CM could be alleviated by employing another strong constitutive promoter, we generated additional αPIG-iPSC lines in which FLuc expression is driven by a synthetic CAG-promoter which was previously shown to yield the highest levels of transgene expression among eight different promoters tested after transient transfection of murine ESC [37]. The extracellular portion of CD4 receptor was co-expressed with FLuc to provide another method to quantify promoter activity during differentiation (**Figure 2A**). Consistent with the UbC-FLuc αPIG-iPSC line, the pCAG-CD4/FLuc αPIG-iPSC lines displayed clonal variability of FLuc intensity in pluripotent state (**Figure 2B**) and a pronounced decrease of BL signal intensity during spontaneous differentiation to 49±50.2% (clone A1) and 15.8±8.2% (clone C4) on day 8 and 26.2±19.5% (clone A1) and 32.5±24.8% (clone C4) at day 16 of differentiation (**Figure 2C**). After purification of CM on day 16 of differentiation, the Fluc activity decreased even further in clone A1 and slightly increased in clone C4 (**Figure 2C**). The pronounced expression of the CAG-driven CD4-receptor on the surface of undifferentiated iPSC rapidly declined in differentiated EB cells and in purified CM, mirroring the reduction of FLuc activity in these clonal cell lines (**Figure 2D,E**). Comparable decline of FLuc activity in the course of differentiation was also observed in murine αPIG-ESC lines in which FLuc expression was under the control of a constitutive PGK promoter (**Figure S3D** in File S1). This was accompanied by a similar decrease of FLuc transcript levels in differentiated cells (**Figure S3E** in File S1). These data indicate that significant transgene silencing occurs during differentiation of murine iPSC and ESC independently of the promoter used or the nature of the expressed transgene, and that a different approach for genetic modification of pluripotent stem cells is required to overcome epigenetic repression of transgene expression.

### Transgene insertion into ROSA26 locus reduces FLuc silencing in iPS-CM

**Figure 3:**
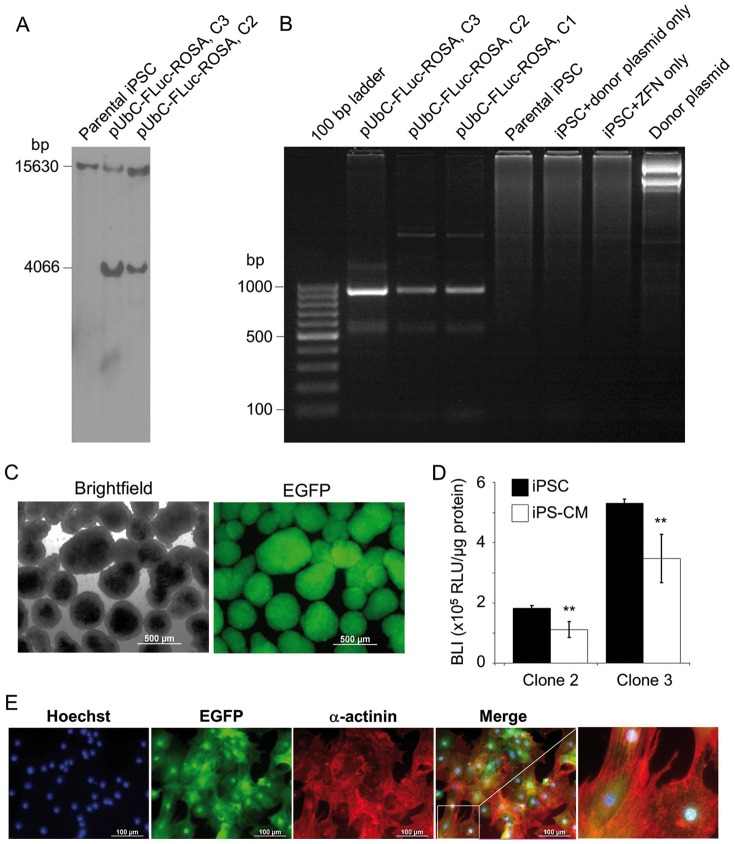
Generation of pUbC-FLuc-ROSA αPIG-iPSC. **A.** Genomic DNA was isolated from indicated cell lines, digested with *Eco*RI and hybridized with a probe for ROSA26 locus in a Southern blot analysis. Expected size of the wild type allele is 15630 bp and of successfully targeted allele 4066 bp because the integration of FLuc cassette results in an additional *Eco*RI restriction site. Presence of both bands in transgenic clones indicates heterozygous integration. **B.** PCR amplification of genomic DNA isolated from indicated cell lines and donor plasmid to verify the site specific integration of FLuc-expression cassette into the ROSA26 locus with an expected PCR product size of 950 kb. **C.** Representative brightfield and fluorescent images of puromycin-purified day 16 pUbC-FLuc-ROSA αPIG-iPSC-derived EGFP positive cardiac clusters. Scale bar  = 500 µm. **D.** Relative luminescence units (RLU) per µg protein in the two clones (C3 and C5) of undifferentiated pUbC-FLuc-ROSA αPIG-iPSC and CM derived from them on day 16 of differentiation. Values are from triplicate measurements of three independent differentiations. Statistics: two-tailed paired Student's t-test. **p<0.01. **E.** CM derived from pUbC-FLuc-ROSA αPIG-iPSC on day 16 of differentiation express EGFP (green) and cardiac structural protein α-actinin (red). Nuclei are stained with Hoechst 33342 (blue). Scale bar: 100 µm.

To avoid excessive down-regulation of the FLuc transgene, we generated a FLuc-αPIG-iPS cell line using ZFN-targeted insertion of the reporter gene driven by UbC-promoter into the silencing-resistant locus ROSA26 [38]. Insertion of the construct into the target region was confirmed by Southern blot analysis. Besides the 15630 bp wild-type fragment, the insertion of the FLuc-cassette was verified by appearance of the 4066 bp targeted DNA fragment using the external ROSA26 probe (**Figure 3A** and **Figure S1** in File S1). The correct insertion of the pUbC[*luc2*/Hygro] cassette into the ROSA26 locus was also confirmed by PCR, using genomic DNA as a template and primers binding in the insert and the genomic region downstream of the target integration site (**Table S1** in File S1), yielding a 950 bp product that was detected in clones 1, 2 and 3 of transgenic pUbC-FLuc-ROSA iPSC, but not in parental iPSC, iPSC transfected only with donor plasmid or ZFN-expression plasmids, and in reactions containing pure donor plasmid (**Figure 3B**). The pUbC-FLuc-ROSA αPIG-iPSC differentiated into GFP-positive CM expressing cardiac α-actinin and exhibiting cross-striations typical of immature CM (**Figure 3C,E**). The assessment of the UbC-driven FLuc activity in ZFN-engineered αPIG-iPSC clones 2 and 3 showed that the FLuc activity decreased, respectively, by 38.9% and 34.3% in antibiotic selected iPS-CM relative to that in pluripotent cells (**Figure 3D**). However, this decrease was significantly lower than that observed in iPS-CM derived from pUbC-FLuc iPSC that were generated by random transgene integration, which lost more than 90% of FLuc activity found in undifferentiated pUbC-FLuc iPSC (see **Figure 1F**). These data suggest that integration of foreign genes into the ROSA26 locus significantly reduces transgene silencing, which may enhance the sensitivity of detection of transplanted cells *in vivo*.

### BL intensity is dependent on the cell line, cell dose and the site of cell injection

**Figure 4:**
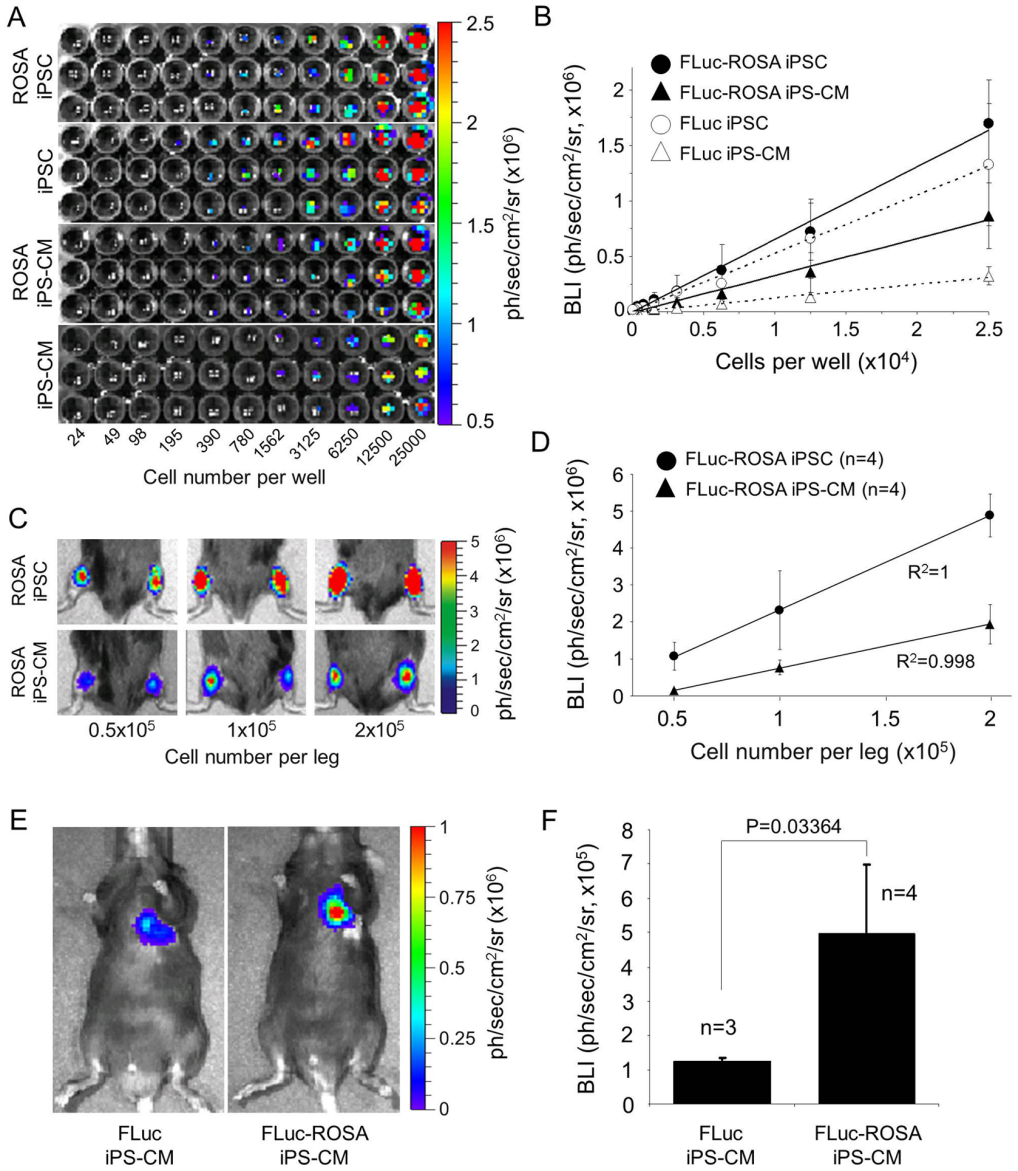
BL signal intensity of FLuc-iPSC and FLuc-iPS-CM depends on the cell dose, genomic locus of transgene integration and site of transplantation. **A.** Optical signal intensity of serial dilutions of 2.5×10^4^ undifferentiated iPSC and purified iPS-CM expressing FLuc from random loci or ROSA26 locus were measured in IVIS. Images are representative results of one serial dilution in triplicate for each cell type. **B.** Demonstration of linearity between cell dose and BL intensity (BLI) of cells shown in panel A. The data are given as mean ± SD of BLI for each cell dose in photons/second/cm^2^/steridian. R^2^ values: FLuc-ROSA-iPSC: 0.9942; FLuc-ROSA-iPS-CM: 0.9876; FLuc-iPSC: 0.9962; FLuc-iPS-CM: 0.9913. **C,D.** Linear relationship between BL signal intensity and cell dose after transplantation of indicated numbers of pUbC-FLuc-ROSA-iPSC and their purified CM into the mouse hind limb muscle. Representative BL images of transplanted mice are shown in panel C and quantitative analysis of these data is shown in panel D as mean ± SD (n = 4) of BLI. **E.** Representative BL images of mice that received intramyocardial injections of 5×10^5^ pUbC-FLuc-iPS-CM or pUbC-FLuc-ROSA-iPS-CM. BL imaging was performed 6 hours after cell injection. **F.** Quantitative analysis of BLI in this experiment is given as mean ± SD for the indicated number of mice. Statistical analysis: two-tailed paired Student's t-test.

One of the prerequisite for accurate optical *in vivo* imaging of transplanted cells is a linear relationship between the BL intensity and the cell dose. *In vitro* comparative BL imaging of serial dilutions of undifferentiated pUbC-FLuc and pUbC-FLuc-ROSA αPIG-iPSC and subsequently purified CM revealed a linear correlation between the BL intensities and cell numbers of both cell types and cell lines (**Figure 4A,B**). In these experiments, undifferentiated FLuc-ROSA-iPSC exhibited slightly higher (22%) levels of BL intensity than FLuc-iPSC. Both iPSC populations emitted stronger BL signal than their purified CM. In agreement with the reduced level of silencing of FLuc integrated into the ROSA26 locus the decrease of the BL signal intensity was much less pronounced in FLuc-ROSA-iPS-CM (2.13±0.17 fold) than in FLuc-iPS-CM (4.43±0.63 fold), relative to their undifferentiated counterparts (**Figure 4B**). Accordingly, purified FLuc-ROSA-iPS-CM showed 2.5±0.2-fold higher FLuc activity than purified FLuc iPS-CM (**Figure 4B**). In order to validate the dose dependency and detection sensitivity *in vivo*, defined numbers of FLuc-ROSA iPSC and puromycin selected FLuc-ROSA iPS-CM were transplanted into the hind limb muscle of mice. This site was selected due to the simplicity of the transplantation procedure compared to open heart surgery and higher efficiency of cell delivery into skeletal muscle as opposed to highly contracting myocardium. BL measurement 6 h after transplantation revealed a linear correlation between optical signal intensity and a number of transplanted FLuc-ROSA iPSC and iPS-CM (**Figure 4C,D**). Although 1×10^5^ and 2×10^5^ transplanted iPS-CM generated, respectively, 3.0- and 2.5-fold weaker BL signal than injected iPSC they were still detectable at high sensitivity. Intramyocardially injected FLuc-ROSA iPS-CM (5×10^5^/heart) produced at 6 h after transplantation a BL signal that was about 7–10-fold weaker than that produced by the same number of CM injected into the skeletal muscle (compare **Figure 4D and 4F**). However, FLuc-ROSA-iPS-CM injected into the heart showed about 4-fold higher BL signal intensity than FLuc-iPS-CM (**Figure 4E,F**), confirming much higher *in vivo* detection sensitivity of CM expressing FLuc from ROSA26 locus compared to those expressing FLuc from random loci.

### Survival kinetics of CM transplanted into the cryoinfarcted heart

**Figure 5:**
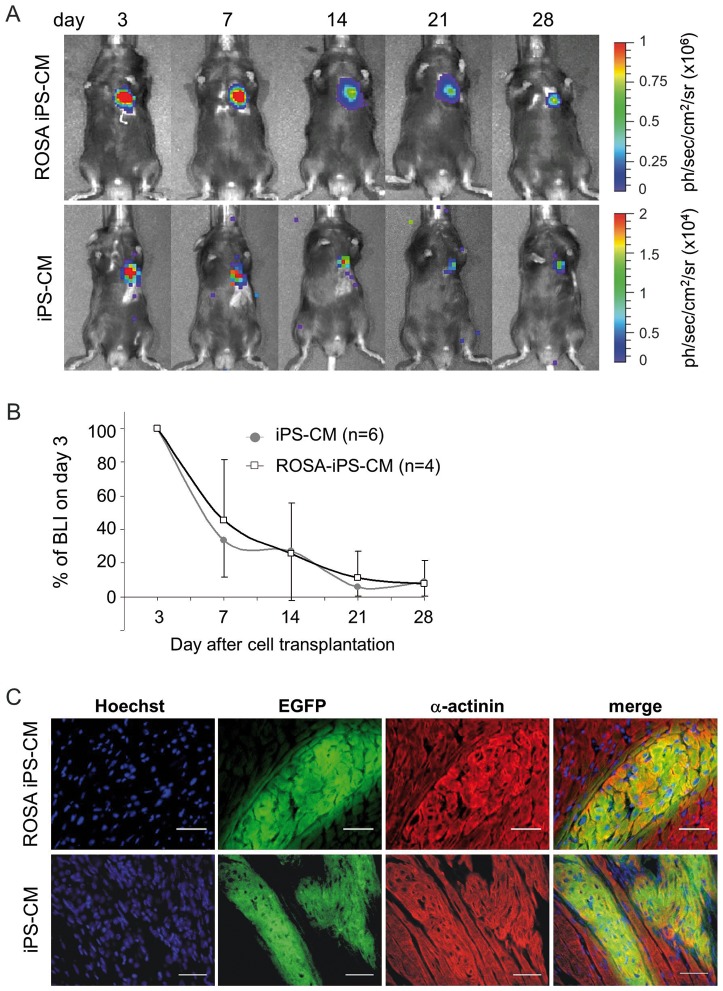
BL imaging of iPS-CM survival over 28 days after transplantation into the cryoinjured heart. **A.** Representative images of mice injected with 5×10^5^ purified pUbC-Fluc-iPS-CM or pUbC-FLuc-ROSA-iPS-CM into the cryoinjured heart. BL measurements were performed on day 3, 7, 14, 21 and 28 after cell injection. **B.** Quantitative analysis of BL signal intensity (BLI) given as mean ± SD of percentages of values relative to BLI on day 3 after cell transplantation. **C.** After four weeks, the experiments were terminated and heart tissue analyzed by immunohistochemistry for the presence of EGFP-positive (green) and α-actinin-positive (red) FLuc-iPS-CM. Transplanted cells were found only in healthy myocardium adjacent to infarct area. Nuclei were counterstained with Hoechst 33342 (blue). Scale bars: 50 µm.

To assess the kinetics of CM retention *in vivo*, 5×10^5^ purified pUbC-FLuc-ROSA iPS-CM and pUbC-FLuc iPS-CM were injected into the periinfarct area of a cryoinjured heart of syngeneic C57/BL6 mice. BL intensity was measured between day 0 and day 28 after transplantation (**Figure 5A**; **Figure S4** in File S1). The BL signal intensity of transplanted FLuc-ROSA iPS-CM or FLuc-iPS-CM dropped 24 hours after transplantation to 44.7±9.4% and 29.2±7.2% of the value obtained on day 0, respectively, and then increased again on postoperative day 3 in the absence of significant CM proliferation (**Figure S4** in File S1). Due to difficulty in obtaining stable baseline signal intensity during the first day after cell injection (which was also reported by others [25], [39]), we performed normalization of signal intensities obtained on days 7, 14, 21 and 28 after transplantation according to signal detected on day 3 postinjection. Using this approach to determine the kinetics of CM retention, we found that the optical signal on day 7 after transplantation decreased to 45±36% in FLuc-ROSA iPS-CM and 34±22% in FLuc iPS-CM compared to the BL intensity in the corresponding group on day 3 (**Figure 5B**). The signal intensity decreased further at a slower rate in both experimental groups and reached on postoperative day 28 about 8% of day 3 signal intensity. After termination of the experiments, heart tissue was histologically analyzed for the presence of EGFP-positive cells. This analysis revealed EGFP-positive areas in the peri-infarct area of both FLuc-ROSA-iPS-CM- and FLuc-iPS-CM-transplanted hearts. Staining of these sections for the sarcomeric α-actinin showed that the transplanted CM possess typical cross-striations which show a tendency of alignment with the surrounding host tissue (**Figure 5C**). However, neither the scar size nor the capillarization in the peri-infarct area was affected by CM transplantation (**Figure S5** and **Supplemental data** in File S1), presumably due to an insufficient number of retained cells. We did not observe presence and outgrowth of any other cell types or indications of teratocarcinoma formation in any of the animals injected with purified FLuc-iPS-CM. These data demonstrate that the majority of iPS-CM are lost in the cryoinjured heart within the first few days post transplantation. However, despite this initial cell loss a small fraction of injected CM is able to survive until day 28 at numbers insufficient to bring about a measurable therapeutic benefit.

## Discussion

Low cell retention after transplantation into infarcted heart is the major obstacle to successful clinical translation of cell-based heart repair approaches. This problem occurs independently of the transplanted cell type, the site of transplantation or the delivery method. Therefore, it is of major importance to address this problem in order to improve the engraftment and enhance the therapeutic efficacy of transplanted cells. BL imaging using FLuc is a powerful method for the quantitative assessment of cell survival *in vivo* and has frequently been used for longitudinal monitoring of various types of intramyocardially transplanted cells in small animals including adult stem cells [16], [40]–[46], fibroblasts [16], embryonic cardiomyoblasts [26], [47], [48], cardiac stem cells [24], [49]–[51], undifferentiated ESC [52], [53], ESC-derived endothelial cells [54] and ES-CM [22], [23], [31], [55]. In this study we describe the generation of transgenic murine iPSC lines in which constitutively active FLuc and CM-specific PAC and EGFP expression allow for isolation, longitudinal *in vivo* bioluminescent tracking and *in situ* fluorescent detection of highly purified iPS-CM. We established 2 stably transgenic pUbC-FLuc iPSC clones that maintained high BL activity in long-term culture in an undifferentiated state. However, the expression of a pUbC-driven FLuc transgene was largely reduced in these iPSC after inducing differentiation by EB formation as well as in purified iPS-CM. This phenomenon was also observed in transgenic murine iPSC lines in which the FLuc and CD4 receptor expression were under the control of a composite CAG promoter. A similar problem was also encountered when FLuc was expressed from the constitutive PGK promoter in a murine αPIG ESC line. These observations are in agreement with previous reports about transgene silencing in stem cells and their differentiated derivatives that was attributed to extensive DNA methylation of promoter sequences and histone modifications [56]–[60].

Epigenetic silencing of exogenous sequences is a major obstacle to efficient transgene expression in pluripotent stem cells and their differentiated derivatives, and negatively impacts their use for studying stem cell differentiation, engraftment and disease modelling. In order to protect transgenes from being silenced, sequences which actively prevent silencing, such as sea urchin insulator [61], cHS4 chromatin insulator elements [57], ubiquitous chromatin opening elements [58] and epigenetic human Matrix Attachment Region (MAR) control elements have been incorporated into the expression vectors and are shown to block transgene silencing in engineered pluripotent stem cell lines. However, due to random integration of such vectors into the host genome, this approach is still not ideal to obtain genetically intact and homogenous reporter cell lines. New technologies for site-specific transgene insertion by using zinc finger nucleases [62] or transcription activator-like effector nucleases (TALEN) [63] are capable of integrating transgenes into genomic regions in which silencing is greatly reduced or eliminated [49], [55]. Indeed, we achieved a significant reduction of silencing of FLuc in iPS-CM by integrating the pUbC-driven FLuc cassette into the ROSA26 locus using ZFN-based genome editing. However, even in this scenario FLuc activity decreased after differentiation to CM. Although this may have occurred, at least in part, due to differential regulation of promoter activity in iPSC and CM, choosing the appropriately designed promoter and vector sequences may still be crucial to enable strong and stable transgene expression in pluripotent stem cell derivatives [37], [57]. Nevertheless, purified iPS-CM were readily detectable *in vivo* after cardiac transplantation producing a robust BL signal that was about 4-fold higher in hearts with transplanted FLuc-ROSA iPS-CM compared to iPS-CM carrying randomly integrated FLuc transgene, reflecting different degree of FLuc silencing during differentiation of these two iPSC lines. The *in vivo* BL signal of injected FLuc-ROSA-iPS-CM was about 10-fold higher when cells were transplanted into the hind leg skeletal muscle than into the heart. These results most likely reflect the differential rate of mechanical cell loss in these two organs immediately upon injection, and/or a more pronounced attenuation of a BL signal emitted from deeper tissue layers in infarcted heart than from more superficially located healthy hind leg muscle. FLuc-expressing iPS-CM retained structural and functional characteristics typical of native iPS-CM [20], [64]. This is in agreement with previously published reports demonstrating that various stable reporter proteins did not significantly alter characteristics of undifferentiated ESC and CM derived from them [23], [49], [65], [66], indicating that BL imaging is a suitable method for non-invasive and longitudinal *in vivo* imaging of cardiac ESC and iPSC derivatives. The *in vivo* optical signal of both iPS-CM populations analyzed in this study decreased rapidly in the first week post-injection and continued to drop at a slower rate until the end of the observation period. This CM survival kinetics did not seem to be affected by the genomic site into which the FLuc transgene was integrated. The presence of small areas of surviving iPS-CM in the peri-infarct area of myocardium on day 28 day post transplantation demonstrates that a fraction of the transplanted iPS-CM is capable of long-term survival. Therefore, in order to improve cell retention methods should be developed to prevent the rapid cell loss early after cell injection and increase the long-term survival of retained cells.

Our observations about the rapid iPS-CM loss are in agreement with those reported for intramyocardially transplanted ES-CM and other cell types. Wu and coworkers were the first group demonstrating the usefulness of BL imaging for monitoring the location and survival of intramyocardially transplanted cells in living rats by employing embryonic rat H9c2 cardiomyoblasts expressing the Fluc reporter gene [26]. A drastic reduction of the BL signal intensity within the first 4 days after transplantation was observed with less than 10% of initial BL signal intensity remaining on day 16 after transplantation, comparable to our findings. The first study that assessed the survival of partially purified human ES-CM showed that more than 90% of cells died within the first 21 days after delivery into the ischemic myocardium of SCID mice [23]. Other studies investigated the retention of bioluminescent adult stem cells [67], cardiac stem cells [49], human ESC-derived endothelial cells [55] and purified human ES-CM [22], [55] consistently reported rapid loss and low survival of cells transplanted into the ischemic heart, irrespective of the genetic background of the recipient, the transplanted cell type and their proliferation rate, suggesting that common factors negatively affect the survival of various cell types in the heart.

A number of factors influence acute and long-term cell retention. Immediately after direct intramyocardial injection a large proportion of cells is lost due to mechanical leakage from the injection site, facilitated by the contracting myocardium and local bleeding [25]. These factors are most likely responsible for a rapid loss of iPS-CM in the first minutes and hours after injection. However, these early effects are difficult to analyze with BL imaging due to unpredictable BL signal variability in the first 24 hours post cell injection [25]. It was recently reported that the enzymatic dissociation of the cells and the transplantation vehicle significantly interfere with the cellular energy metabolism resulting in a survival-independent bias of BL-signal obtained during the first 24 hours after transplantation due to its dependence on ATP availability [39]. We have also encountered unexpected fluctuations of BL signal intensity in the first 48 hours post cell transplantation, and this is why we have chosen to analyse the CM survival kinetics from postoperative day three onwards. At that time the cell energy metabolism was expected to have recovered from enzymatic dissociation and oxygen and nutrient deprivation in the cardiac tissue disrupted by infarction and the injection needle.

Among other factors that diminish cell retention after transplantation are lack of integration into the host tissue and apoptosis due to adverse effects of tissue microenvironment, inflammation and immune rejection at the site of injection. We have previously shown that dissociated murine ES-CM were not capable of forming secondary cardiospheres in hanging drops and attaching quantitatively to any of 16 tested 2-dimensional biomaterials [68], indicating that purified CM have highly selective requirements for cell-to-cell or cell-to-extracellular matrix interactions, which may not be available in the damaged myocardium early upon injection. Various approaches have been tested to enhance the survival of transplanted cells in the myocardium. These include the preconditioning of cells prior to injection with various pro-survival factors (cytokines, growth factors, drugs, microRNAs) [50], [69] or physical stimuli (hypoxia, heat shock) [70], [71], transplantation of cell sheets [72] or cells embedded in tissue-engineered 3D matrices [47], instead of direct injection of enzymatically dissociated cells. In addition, genetic engineering of cells to stably express pro-survival or angiogenic factors [51], [73] and use of more immature cells for transplantation that display higher proliferation rates and increased hypoxia resistance than mature cells [22] were used. Finally, co-transplantation of supportive cell types, such as mesenchymal stromal cells, that may increase the engraftment of primary therapeutic cells by providing anti-inflammatory, anti-apoptotic and pro-angiogenic stimuli [73], [74] represents an especially interesting approach. The FLuc expressing ZFN-engineered iPS-CM generated in this study will enable further optimization of combined approaches for improving the survival, integration and therapeutic efficacy of iPS-CM as a highly accessible *in vitro* source of cells for heart repair.

## Supporting Information

File S1 **Figure S1**, Targeted integration of a firefly luciferase reporter gene into the ROSA26 locus. Structures of targeting vector, wild-type ROSA26 locus and ZFN-targeted ROSA26 locus are shown. Targeting vector pDonor-pUbC [*luc2*/Hygro]-ROSA26 was used for insertion of the transgene cassette into the ROSA26 locus by homology-directed repair of the double-strand brake induced by ZFN encoded by plasmids pCMV-RosaL6 and pCMV-RosaR4. The targeting construct contains the UbC promoter driving constitutive expression of FLuc gene and SV40-promoter driving expression of a selectable marker for hygromycin resistance flanked by the left (796 nt) and right (815 nt) homology arms. The structure of the wild-type ROSA26 locus depicts the ZFN^Rosa^ cleavage site within an intronic *Xba*I site (arrowhead). Small open boxes indicate exon regions. The location of *Eco*RI sites and Southern blot probe located in the ROSA26 locus upstream of the transgene integration site are indicated. The sizes of *Eco*RI restriction fragments of wild-type allele and targeted ROSA26 allele that can be detected with the Southern blot probe are 15630 nt and 4066 nt, respectively. Forward PCR primer (F) binding to targeting vector and reverse primer (R) binding to ROSA26 locus downstream of the integration site were used to amplify a 950 nt diagnostic fragment and are indicated by black arrowheads. **Figure S2**, FLuc-αPIG-iPSC-derived CM are structurally and functionally intact. **A.** Flow cytometric analysis of puromycin selected FLuc-αPIG -iPS-CM (clone # C3) at day 16 of differentiation. Left panel: gated cell population (2×10^4^ events) on forward and sideward scatter dot plot. Right panel: dot plot showing the distribution of propidium iodide (PI)-positive cells and EGFP-positive viable iPS-CM in the gated cell population. **B.** Semiquantitative RT-PCR analysis for indicated cardiac specific transcripts in purified FLuc-αPIG-iPS-CM (clone # C3, upper panel) and parental iPSC-derived CM (lower panel). **C.** Immunofluorescence detection of EGFP, α-actinin 2 and cardiac troponin T (cTnT) in purified FLuc-αPIG -iPS-CM (clone # C3) plated on fibronectin-coated dishes. Nuclei were stained with DAPI. Scale bar: 20 µm, magnifications were enhanced 2.15-fold. **D.** Representative action potentials (AP) of spontaneously beating FLuc-αPIG-iPS-CM recorded by the whole-cell current-clamp technique before, during and after wash-out (WO) addition of 1 µM isoproterenol (Iso) and 1 µM carbachol (Cch). **E.** Quantitative analysis of beating rates of FLuc-αPIG-iPS-CM before, during and after (wash-out, WO) addition of Iso or Cch. Data are given as mean ± SD (n = 5 cells in each group; **p<0.01; ***p<0.001). **Figure S3**, Generation and characterization of Fluc-activity in pPGK-FLuc αPIG-ESC lines. **A.** Schematic representation of the pGL4.14-pPGK [*luc2*/Hygro] plasmid used to generate transgenic pPGK-FLuc-αPIG-ESC lines. **B.** Bioluminescence signal intensity (BLI) in 17 pPGK-FLuc ESC clones obtained after hygromycin selection. Values represent relative luminescence units (RLU) in live cell measurements of 1×10^6^ cells/well in a single experiment. **C.** Linear relationship between cell dose and BLI of pPGK-FLuc ESC clone F2. Data are given as mean ± SD of triplicate measurements. **D.** Four pPGK-FLuc-αPIG-ESC clones with highest FLuc-activity were subjected to spontaneous in vitro differentiation. BLI was measured in undifferentiated ESC, cells from dissociated day 6 and day 16 EB and in puromycin-selected cardiomyocytes on day 16 of differentiation (d16 CM) in the GENios Pro microplate reader. Data are given for a single measurement of 5×10^5^ cells/well of a 96-well plate. **E.** Relative FLuc transcript levels in undifferentiated ESC, day 16 EB and purified day 16 cardiomyocytes (d16 CM) of pPGK-FLuc ESC clones B2 and B5 as determined by RT-qPCR. Note the dramatic reduction of FLuc mRNA expression in differentiating EB cells and purified CM. Data were normalized to GAPDH as a housekeeping gene control and are given as mean ± SD of triplicate measurements. **Figure S4**, Significant decline of the bioluminescence signal intensity on the first day after transplantation of FLuc-expressing iPSC-derived cardiomyocytes. 5×10^5^ purified pUbC-FLuc iPS-CM or pUbC-FLuc-ROSA iPS-CM were transplanted into the cryoinjured heart of syngeneic mice (n = 4) and BL measurements were performed on day 0, day 1 and day 3 after cell injection. Data are shown relative to BL signal intensity that was determined on day 0 six hours after CM transplantation. Statistical analysis of day 0 *versus* day 1 and day 1 *versus* day 3 BL intensities was performed using the two-tailed paired Student's t-test. p-values for pUbC-FLuc-ROSA and pUbC-FLuc iPS-CM are shown on upper and lower lines, respectively. The pattern of BL fluctuation in mice injected with pUbC-FLuc-ROSA iPS-CM was similar to that observed in pUbC-FLuc iPS-CM-transplanted mice but did not reach statistical significance due to a large coefficient of variation within the former group on day 0 and day 3. **Figure S5**, No significant effect on capillary density and fibrotic area four weeks after transplantation of iPS-CM into cryoinjured hearts. **A.** Scar size was evaluated by Masson's trichrome (MTC) staining of histological sections of hearts four weeks after cryoinfarction and injection of either PBS saline (sham group) or iPS-CM. n = 5 animals for both groups, p = 0.118. **B.** Representative images of MTC stained sections of sham and iPS-CM treated hearts. **C.** Capillarization in periinfarct region of sham and iPS-CM-transplanted hearts was assessed by calveolin staining (green fluorescence). n = 5 animals for both groups, p = 0.0832. **D.** Representative calveolin stained sections of sham and iPS-CM treated hearts. Scale bars: 50 µm. Selected areas in panels B and D are shown magnified in the corresponding right hand panels.
(DOC)
