# Supplementary material for: Bioluminescent Imaging of Genetically Selected Induced Pluripotent Stem Cell-Derived Cardiomyocytes after Transplantation into Infarcted Heart of Syngeneic Recipients
Source: PLoS One. 2014 Sep 16;9(9):e107363. doi: 10.1371/journal.pone.0107363 (PMC4167328; doi:10.1371/journal.pone.0107363)
Supplement: File S1 — Figure S1, Targeted integration of a firefly luciferase reporter gene into the ROSA26 locus. Structures of targeting vector, wild-type ROSA26 locus and ZFN-targeted ROSA26 locus are shown. Targeting vector pDonor-pUbC [luc2/Hygro]-ROSA26 was used for insertion of the transgene cassette into the ROSA26 locus by homology-directed repair of the double-strand brake induced by ZFN encoded by plasmids pCMV-RosaL6 and pCMV-RosaR4. The targeting construct contains the UbC promoter driving constitutive expression of FLuc gene and SV40-promoter driving expression of a selectable marker for hygromycin resistance flanked by the left (796 nt) and right (815 nt) homology arms. The structure of the wild-type ROSA26 locus depicts the ZFNRosa cleavage site within an intronic XbaI site (arrowhead). Small open boxes indicate exon regions. The location of EcoRI sites and Southern blot probe located in the ROSA26 locus upstream of the transgene integration site are indicated. The sizes of EcoRI restriction fragments of wild-type allele and targeted ROSA26 allele that can be detected with the Southern blot probe are 15630 nt and 4066 nt, respectively. Forward PCR primer (F) binding to targeting vector and reverse primer (R) binding to ROSA26 locus downstream of the integration site were used to amplify a 950 nt diagnostic fragment and are indicated by black arrowheads. Figure S2, FLuc-αPIG-iPSC-derived CM are structurally and functionally intact. A. Flow cytometric analysis of puromycin selected FLuc-αPIG -iPS-CM (clone # C3) at day 16 of differentiation. Left panel: gated cell population (2×10^4^ events) on forward and sideward scatter dot plot. Right panel: dot plot showing the distribution of propidium iodide (PI)-positive cells and EGFP-positive viable iPS-CM in the gated cell population. B. Semiquantitative RT-PCR analysis for indicated cardiac specific transcripts in purified FLuc-αPIG-iPS-CM (clone # C3, upper panel) and parental iPSC-derived CM (lower panel). C. Immunofluorescence [file pone.0107363.s001.doc]

Supporting Information File S1

Bioluminescent imaging of genetically selected induced pluripotent stem cell-derived cardiomyocytes after transplantation into infarcted heart of syngeneic recipients

Vera Lepperhof^1^, Olga Polchynski^1^, Klaus Kruttwig^2^, Chantal Brüggemann^2^, Klaus Neef^3,4^, Florian Drey^3^, Yunjie Zheng^1^, Justus P. Ackermann^5^, Yeong-Hoon Choi^3,4^, Thomas F. Wunderlich^4,5,6^, Mathias Hoehn^2^, Jürgen Hescheler^1^, Tomo Šarić^1^

*^1^ Institute for Neurophysiology, University of Cologne, Cologne, Germany*

*^2^ In-vivo-NMR Laboratory, Max Planck Institute for Neurological Research, Cologne, Germany*

*^3^ Department of Cardiothoracic Surgery, Heart Center of the University of Cologne, Cologne, Germany*

*^4^ Center for Molecular Medicine Cologne, University of Cologne, Cologne, Germany*

*^5^ Max Planck Institute for Metabolism Research and Institute for Genetics, Cologne, Germany*

*^6^ Cologne Cluster of Excellence in Cellular Stress Responses in Aging-associated Diseases (CECAD), Cologne, Germany*

**Table of Content** page

Supplemental methods …………………………………………. 2

Supplemental data …………………………………………. 4

Supplemental figures …………………………………………. 6

Figure S1 …………………………………………. 6

Figure S2 …………………………………………. 7

Figure S3 …………………………………………. 8

Figure S4 …………………………………………. 9

Figure S5 …………………………………………. 10

Table S1 …………………………………………. 11

**Supplemental methods**

**qPCR and RT-PCR**

Total RNA was isolated from iPSC, EB or cardiac clusters by Trizol Reagent following the manufacturer’s recommendations (Life Technologies). RNA concentration was measured with a Nanodrop 1000 (Thermo Scientific) and the quality was assessed by agarose gel electrophoresis. cDNA samples were synthesized from 2 µg of total RNA using the SuperScript II First-Strand Synthesis Kit (Invitrogen) and random hexamers for priming. qPCR was performed using SYBR advantage qPCR premix (Clontech, Mountain View, CA, USA ), 30 ng cDNA and primers (**Table S1** in this File S1) at a final concentration of 5 pM in a 7500 Fast Real-Time PCR System (Applied Biosystems, Foster City, CA, USA). The results were analyzed with the manufacturer’s software for 7500 Fast thermocyclers version 2.5.0.2. Semi-quantitative RT-PCR was carried out using Dream Taq Mastermix (Thermo Scientific) and PCR products were analyzed by agarose gel electrophoresis with a 100 bp ladder (Thermo Scientific) to determine the PCR product sizes. Primers used in these analyses are listed in Table S1.

**Electrophysiology**

Puromycin-purified cardiac clusters were dissociated into single CM with 0.05% Trypsin/EDTA (Invitrogen) and plated on 0.1% gelatine (Sigma-Aldrich)-coated glass cover slips and cultured for 24-48 hours before measurements. The cover slips were placed into a recording chamber (37°C) and cells perfused continuously with extracellular solution. Cell membrane capacitance was determined on-line using the PULSE software (HEKA Elektronik, Lambrecht Pfalz, Germany). Action potentials (AP) of spontaneously beating CM were recorded by the whole-cell current-clamp technique using an EPC-9 amplifier (HEKA Elektronik) and the PULSE program. Response of CM to hormonal regulation was assessed by administering isoproterenol (Iso) and carbachol (CCh) (Sigma-Aldrich). Data are presented as the mean ± standard error of the mean (SEM) (**Figure S2** in this File S1). Student’s *t* test was applied for statistical evaluation.

**Immunohistochemistry and histology**

After final BL measurements, the mice were euthanized, hearts excised, flushed with PBS, embedded in Tissue-Tek O.C.T. (Sakura Finetek, Staufen, Germany) and snap-frozen in isobutane (2‑methylpropane, Roth) cooled with dry ice and stored at -80°C. Hearts were cryo-sectioned (10 µm) along the short axis (transversal) using a cryotome CM-1950 (Leica, Wetzlar, Germany). For the assessment of capillary density cryosections were fixed using acetone/chloroform and incubated with anti-Caveolin-1 antibody (1:300; Acris, Herford, Germany) and species-matched, fluorescently labeled (FITC, fluorescein isothiocyanate) secondary antibody (1:200; Life Technologies, Darmstadt, Germany). The density of capillaries (events per mm^2^) was calculated automatically from photomicrographs of whole sections using a Nikon Eclipse Ti-U microscope with NIS Elements software v2.4 (both Nikon, Düsseldorf, Germany). For the assessment of infarction scar area sizes, every tenth cryosection was stained using the Masson’s Trichrome kit (Sigma Aldrich, Taufkirchen, Germany) following the manufacturer’s instructions. The length of the fibrotic area (stained in blue) was measured from microphotographs using a Nikon Eclipse Ti-U microscope with NIS Elements software v2.4. The total scar area was calculated from integrating scar lengths with number of sections (x100 µm: 10 µm thickness of section and 90 µm gaps between successive sections).

**Supplemental data**

**FLuc-αPIG-iPSC differentiate into structurally and functionally intact CM**

In order to ensure that constitutive expression of FLuc in iPSC did not alter their differentiation potential and/or properties of purified CM, we used the iPSC clone C3 exhibiting the highest BL activity to assess in more detail its cardiogenic differentiation capacity and structural and functional characteristics of puromycin selected CM. First EB displaying spontaneous contractions occurred on day 7-8 of differentiation, which was comparable to the appearance of beating activity in parental αPIG-iPSC-derived EB. The purity of FLuc-αPIG-iPS-CM after elimination of non-CM by puromycin treatment of EB for 7 days was on average 98.87±0.37% in ten independent differentiations as determined by assessing the fraction of EGFP expressing viable cells using flow cytometry (**Figure S2A** in File S1). The typical yield of pure FLuc-αPIG-iPS-CM after antibiotic selection was 5.6x10^6^±1.1x10^6^ CM (n = 9) from 1x10^6^ of input iPSC subjected to differentiation. Transcripts encoding for cardiac structural proteins α-actinin (ACTN2), α-myosin heavy chain (MYH6), cardiac troponin T (TNNT2), and transcription factors GATA4 and Nkx2.5 were all expressed in FLuc-αPIG-iPS-CM and in CM derived from the parental αPIG-iPSC line (**Figure S2B** in File S1). Structural integrity of purified FLuc-αPIG-iPS-CM was analyzed by immunocytochemistry (**Figure S2C** in File S1). Cardiac troponin T (cTnT) and α-actinin were expressed by EGFP-positive CM and formed cross striations typical of CM (**Figure S2C** in File S1). Electrophysiological measurements showed that 85% of all analyzed (n=20) puromycin purified FLuc-expressing CM displayed action potentials characteristic of atrial-like CM with rare cells exhibiting pacemaker-like (10%) and ventricular-like (5%) properties. The cells responded to the adrenergic agonist isoproterenol (Iso) with increased beating frequency, whereas the muscarinergic agonist charbachol (Cch) completely abolished cellular beating activity (**Figure S2D,E** in File S1). Both effects could be reversed by removal of the drugs. These data clearly indicate that over-expression of FLuc did not have any adverse effects on structural or functional properties of transgenic CM.

**Effect of transplanted iPS-CM on scar area and capillary density**

In order to determine whether transplanted iPS-CM exerted beneficial effect on infarcted heart, we measured the scar area and capillary density in histological sections of hearts four weeks after cryoinfarction and injection of either PBS (sham group) or pUbC-FLuc iPS-CM. These analyses revealed that neither the scar size nor the capillarization in the peri-infarct area were affected by CM transplantation (**Figure S5** in File S1), most likely due to an insufficient fraction of CM that were retained for long enough to exert a measurable therapeutic effect.

**Supplemental figures**

**
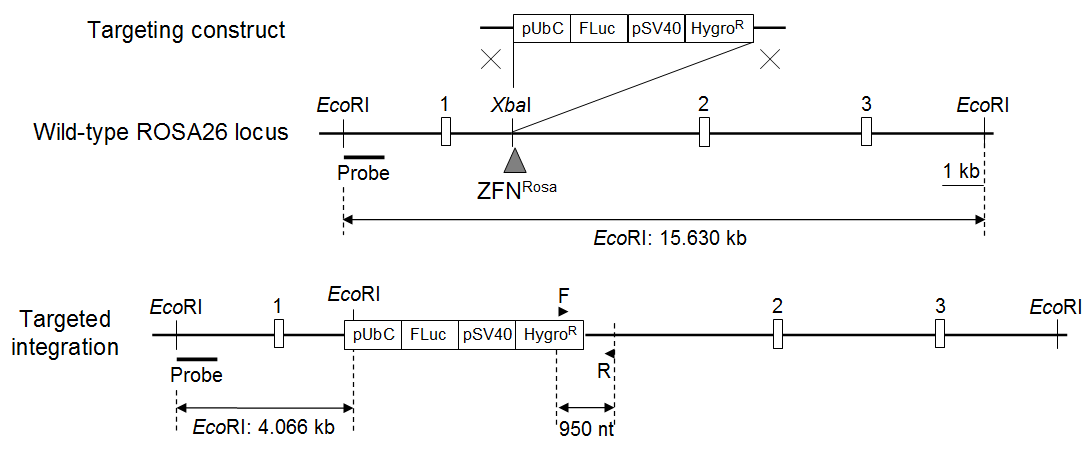
**

**Figure S1. Targeted integration of a firefly luciferase reporter gene into the ROSA26 locus.** Structures of targeting vector, wild-type ROSA26 locus and ZFN-targeted ROSA26 locus are shown. Targeting vector pDonor-pUbC[*luc2/*Hygro]-ROSA26 was used for insertion of the transgene cassette into the ROSA26 locus by homology-directed repair of the double-strand brake induced by ZFN encoded by plasmids pCMV-RosaL6 and pCMV-RosaR4. The targeting construct contains the UbC promoter driving constitutive expression of FLuc gene and SV40-promoter driving expression of a selectable marker for hygromycin resistance flanked by the left (796 nt) and right (815 nt) homology arms. The structure of the wild-type ROSA26 locus depicts the ZFN^Rosa^ cleavage site within an intronic *Xba*I site (arrowhead). Small open boxes indicate exon regions. The location of *Eco*RI sites and Southern blot probe located in the ROSA26 locus upstream of the transgene integration site are indicated. The sizes of *Eco*RI restriction fragments of wild-type allele and targeted ROSA26 allele that can be detected with the Southern blot probe are 15630 nt and 4066 nt, respectively. Forward PCR primer (F) binding to targeting vector and reverse primer (R) binding to ROSA26 locus downstream of the integration site were used to amplify a 950 nt diagnostic fragment and are indicated by black arrowheads.

**
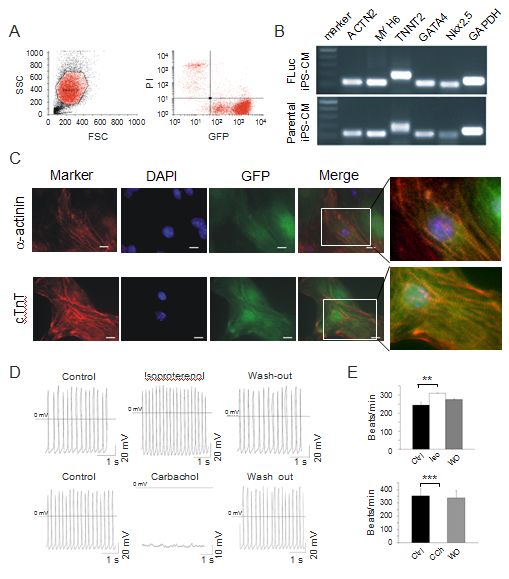
**

**Figure S2. FLuc-αPIG-iPSC-derived CM are structurally and functionally intact.** **A.** Flow cytometric analysis of puromycin selected FLuc-αPIG -iPS-CM (clone # C3) at day 16 of differentiation. Left panel: gated cell population (2x10^4^ events) on forward and sideward scatter dot plot. Right panel: dot plot showing the distribution of propidium iodide (PI)-positive cells and EGFP-positive viable iPS-CM in the gated cell population. **B.** Semiquantitative RT-PCR analysis for indicated cardiac specific transcripts in purified FLuc-αPIG-iPS-CM (clone # C3, upper panel) and parental iPSC-derived CM (lower panel). **C.** Immunofluorescence detection of EGFP, α-actinin 2 and cardiac troponin T (cTnT) in purified FLuc-αPIG -iPS-CM (clone # C3) plated on fibronectin-coated dishes. Nuclei were stained with DAPI. Scale bar: 20 μm, magnifications were enhanced 2.15-fold. **D.** Representative action potentials (AP) of spontaneously beating FLuc-αPIG-iPS-CM recorded by the whole-cell current-clamp technique before, during and after wash-out (WO) addition of 1 µM isoproterenol (Iso) and 1 µM carbachol (Cch). **E.** Quantitative analysis of beating rates of FLuc-αPIG-iPS-CM before, during and after (wash-out, WO) addition of Iso or Cch. Data are given as mean ± SD (n=5 cells in each group; ** p<0.01; *** p<0.001).

**
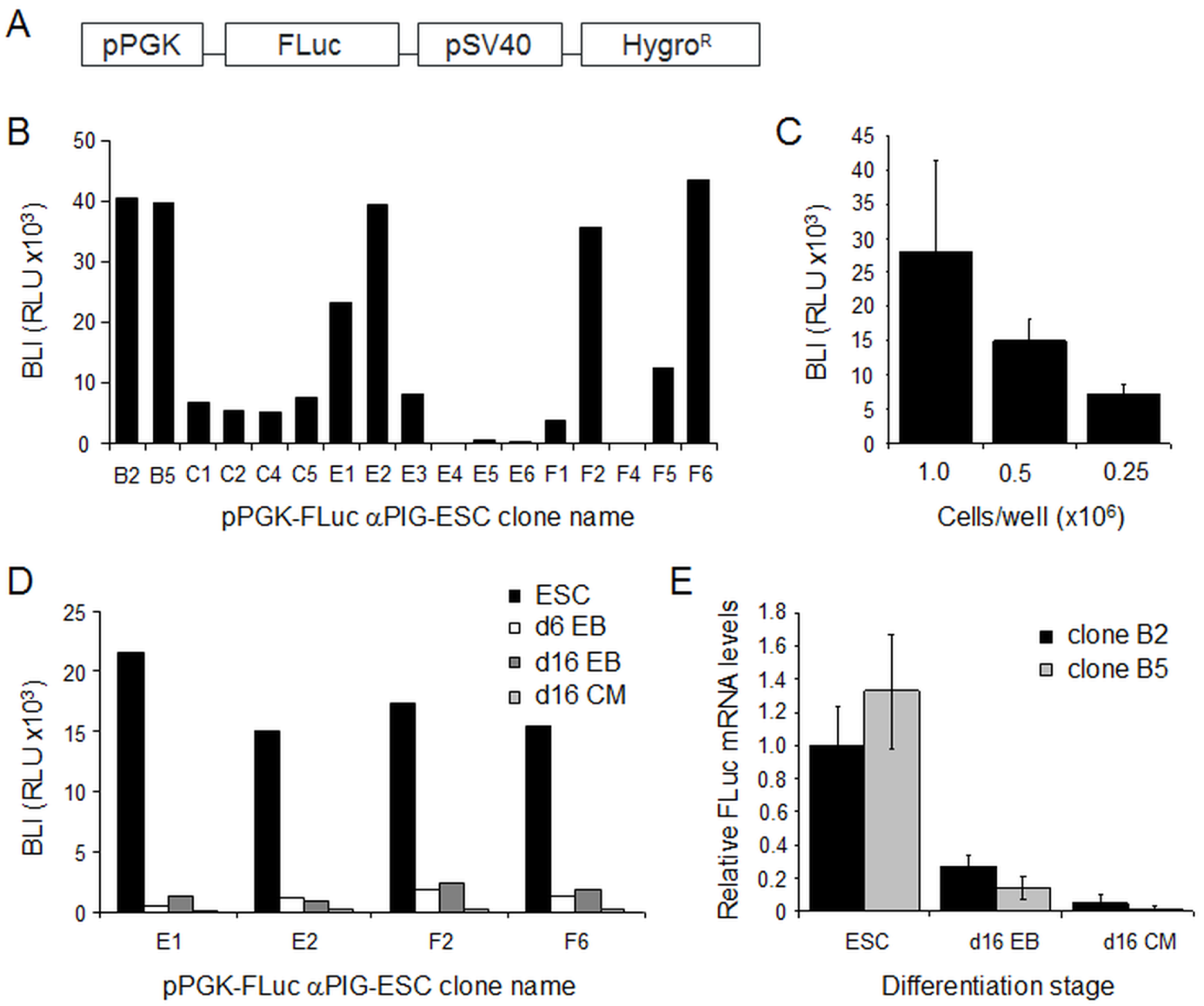
**

**Figure S3. Generation and characterization of Fluc-activity in pPGK-FLuc αPIG-ESC lines. A.** Schematic representation of the pGL4.14-pPGK[*luc2*/Hygro] plasmid used to generate transgenic pPGK-FLuc-αPIG-ESC lines. **B.** Bioluminescence signal intensity (BLI) in 17 pPGK-FLuc ESC clones obtained after hygromycin selection. Values represent relative luminescence units (RLU) in live cell measurements of 1x10^6^ cells/well in a single experiment. **C.** Linear relationship between cell dose and BLI of pPGK-FLuc ESC clone F2. Data are given as mean ± SD of triplicate measurements. **D.** Four pPGK-FLuc-αPIG-ESC clones with highest FLuc-activity were subjected to spontaneous in vitro differentiation. BLI was measured in undifferentiated ESC, cells from dissociated day 6 and day 16 EB and in puromycin-selected cardiomyocytes on day 16 of differentiation (d16 CM) in the GENios Pro microplate reader. Data are given for a single measurement of 5x10^5^ cells/well of a 96-well plate. **E.** Relative FLuc transcript levels in undifferentiated ESC, day 16 EB and purified day 16 cardiomyocytes (d16 CM) of pPGK-FLuc ESC clones B2 and B5 as determined by RT-qPCR. Note the dramatic reduction of FLuc mRNA expression in differentiating EB cells and purified CM. Data were normalized to GAPDH as a housekeeping gene control and are given as mean ± SD of triplicate measurements.

**
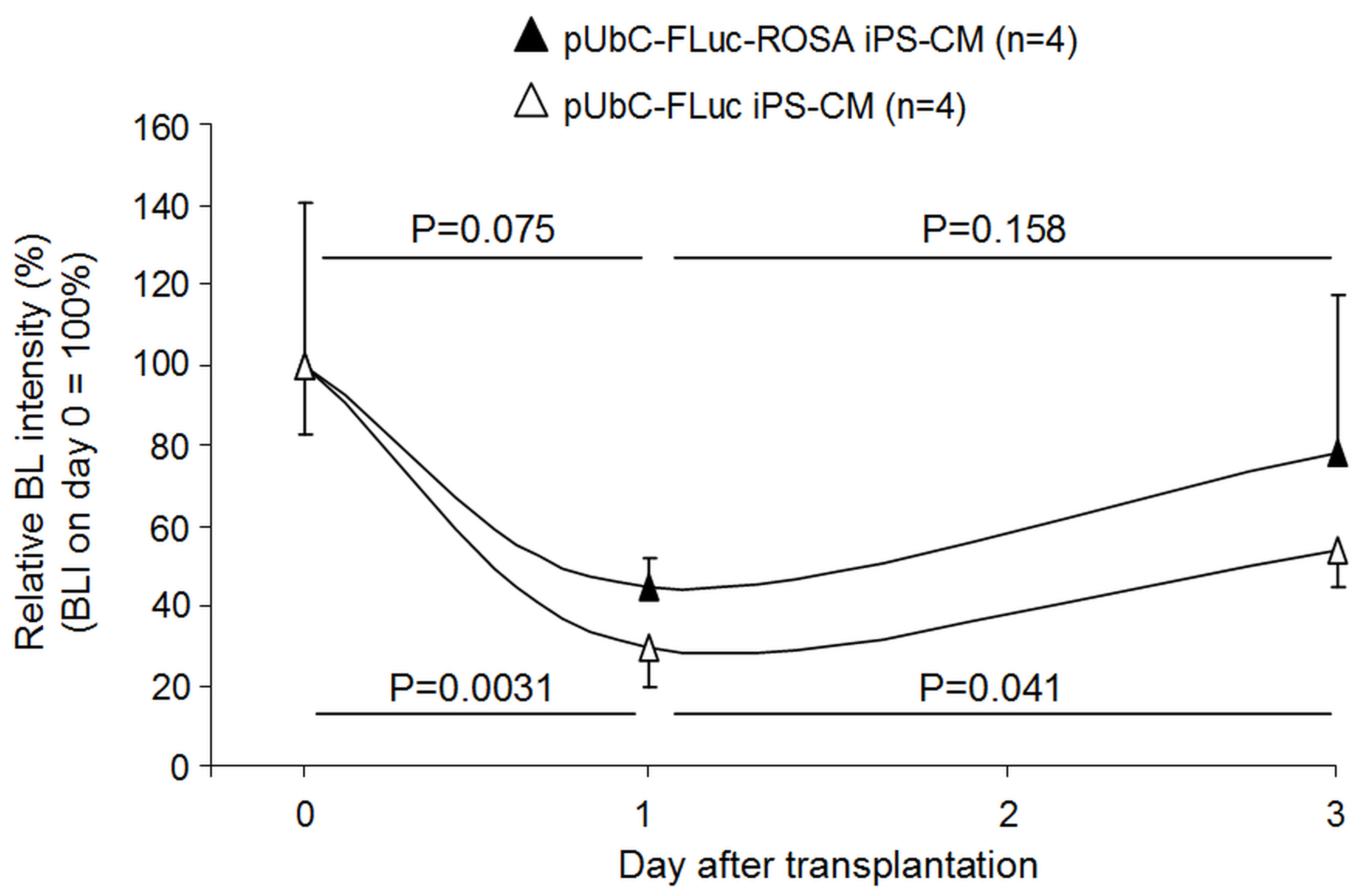
**

**Figure S4. Significant decline of the bioluminescence signal intensity on the first day after transplantation of FLuc-expressing iPSC-derived cardiomyocytes.** 5x10^5^ purified pUbC-FLuc iPS-CM or pUbC-FLuc-ROSA iPS-CM were transplanted into the cryoinjured heart of syngeneic mice (n=4) and BL measurements were performed on day 0, day 1 and day 3 after cell injection. Data are shown relative to BL signal intensity that was determined on day 0 six hours after CM transplantation. Statistical analysis of day 0 *versus* day 1 and day 1 *versus* day 3 BL intensities was performed using the two-tailed paired Student’s t-test. p-values for pUbC-FLuc-ROSA and pUbC-FLuc iPS-CM are shown on upper and lower lines, respectively. The pattern of BL fluctuation in mice injected with pUbC-FLuc-ROSA iPS-CM was similar to that observed in pUbC-FLuc iPS-CM-transplanted mice but did not reach statistical significance due to a large coefficient of variation within the former group on day 0 and day 3.

**
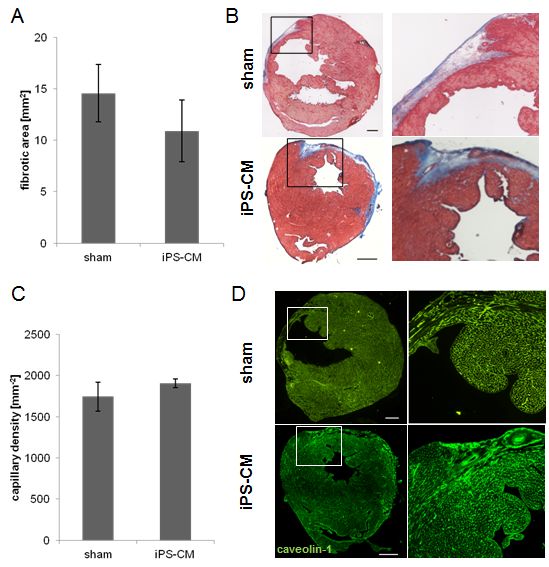
**

**Figure S5. No significant effect on capillary density and fibrotic area four weeks after transplantation of iPS-CM into cryoinjured hearts. A.** Scar size was evaluated by Masson’s trichrome (MTC) staining of histological sections of hearts four weeks after cryoinfarction and injection of either PBS saline (sham group) or iPS-CM. n=5 animals for both groups, p=0.118. **B.** Representative images of MTC stained sections of sham and iPS-CM treated hearts. **C.** Capillarization in periinfarct region of sham and iPS-CM-transplanted hearts was assessed by calveolin staining (green fluorescence). n=5 animals for both groups, p=0.0832. **D.** Representative calveolin stained sections of sham and iPS-CM treated hearts. Scale bars: 50 μm. Selected areas in panels B and D are shown magnified in the corresponding right hand panels.

**Table S1.** **Primers used for RT-PCR and RT-qPCR.**

| **Primer name** | **T_a_ (°C)** | **Sequence**  **(5’-->3’)** | **PCR-product length, nt** | **Vector or gene bank number** |
| --- | --- | --- | --- | --- |
| FLuc_F | 58 | ACTATGTGGCCAGCCAGGTTACAA | 94 | pGL4.14[*luc2/*Hygro] |
| FLuc_R | 58 | TCCAACTTGCCGGTCAGTCCTTTA |  |
| TNNT2_F | 59 | TGGGATGGAGTCAGCGGGCA | 214 | NT_039687.7 |
| TNNT2_R | 59 | AGCCAGGATGGAGCCACCGA |  |
| Nkx2.5_F | 60 | CAGCCAAAGACCCTCGGGCG | 142 | NM_008700.2 |
| Nkx2.5_R | 60 | TGCGCCTGCGAGAAGAGCAC |  |
| GATA4_F | 59 | GAAAACGGAAGCCCAAGAACC | 186 | NM_010861.3 |
| GATA4_R | 60 | TGCTGTGCCCATAGTGAGATGAC |  |
| MYH6_F | 60 | TGTCCCGGGAAGGGGGCAAA | 144 | NM_001164171.1 |
| MYH6_R | 59 | CCGGCTCGTGCAGGAAGGTC |  |
| GAPDH_F | 65 | GGCTCATGACCACAGTCCAT | 142 | NM_008084.2 |
| GAPDH_R | 66 | ACCTTGCCCACAGCCTTG |  |
| Fluc-ROSA_F | 60 | AATATCTTTATTTTCATTACA | 950 |  |
| Fluc-ROSA_R | 60 | GGTAATATTGGGGGAGGAGACATC | 950 |

Abbreviations: F-forward, R-reverse, nt-nucleotides, T_a_ - annealing temperature.
